# Notch modulates VEGF action in endothelial cells by inducing Matrix Metalloprotease activity

**DOI:** 10.1186/2045-824X-3-2

**Published:** 2011-01-18

**Authors:** Yasuhiro Funahashi, Carrie J Shawber, Anshula Sharma, Emi Kanamaru, Yun K Choi, Jan Kitajewski

**Affiliations:** 1 Tsukuba Research Laboratories, Eisai Co., Ltd, Ibaraki, Japan; 2 Pathology and Herbert Irving Comprehensive Cancer Center, Columbia University Medical Center, NY, NY 10032, USA; 3 OB/GYN, Columbia University Medical Center, New York, NY 10032, USA

## Abstract

**Background:**

In the vasculature, Notch signaling functions as a downstream effecter of Vascular Endothelial Growth Factor (VEGF) signaling. VEGF regulates sprouting angiogenesis in part by inducing and activating matrix metalloproteases (MMPs). This study sought to determine if VEGF regulation of MMPs was mediated via Notch signaling and to determine how Notch regulation of MMPs influenced endothelial cell morphogenesis.

**Methods and Results:**

We assessed the relationship between VEGF and Notch signaling in cultured human umbilical vein endothelial cells. Overexpression of VEGF-induced Notch4 and the Notch ligand, Dll4, activated Notch signaling, and altered endothelial cell morphology in a fashion similar to that induced by Notch activation. Expression of a secreted Notch antagonist (Notch1 decoy) suppressed VEGF-mediated activation of endothelial Notch signaling and endothelial morphogenesis. We demonstrate that Notch mediates VEGF-induced matrix metalloprotease activity via induction of MMP9 and MT1-MMP expression and activation of MMP2. Introduction of a MMP inhibitor blocked Notch-mediated endothelial morphogenesis. In mice, analysis of VEGF-induced dermal angiogenesis demonstrated that the Notch1 decoy reduced perivascular MMP9 expression.

**Conclusions:**

Taken together, our data demonstrate that Notch signaling can act downstream of VEGF signaling to regulate endothelial cell morphogenesis via induction and activation of specific MMPs. In a murine model of VEGF-induced dermal angiogenesis, Notch inhibition led to reduced MMP9 expression.

## Background

Angiogenesis, the formation of new blood vessels from existing vasculature, is a multi-step process that plays a central role in embryogenesis and pathological phenomena. Vascular Endothelial Growth Factor (VEGF) is a key regulator of angiogenesis and is important for the degradation of extracellular matrix (ECM), as well as the subsequent proliferation, migration, and survival of endothelial cells. ECM components, including fibrins, collagens, and laminins, form a lamina around existing vasculature that must be degraded in order to form new vessels.

VEGF signaling via VEGFR-2 induces the expression of endothelial cell-derived matrix metalloproteases (MMPs), including MMP2 [1], MMP9 [2], and MT1-MMP [3], which degrade the matrix to allow for endothelial sprouting. MMPs are thus essential for angiogenesis, and their loss from either endothelium or inflammatory cells has been associated with severe angiogenic defects. At the same time, increased MMP activity has significant vascular consequences. MMPs are antagonized by Tissue Inhibitors of Matrix Metalloproteases (TIMPs), and a pathological increase in endothelial MMP over TIMP activity has been proposed to contribute to vessel wall thickening, abdominal aortic aneurysm formation, varicose veins, hypertension and preeclampsia [4-6]. MMPs are not, however, strictly proangiogenic; other more recently elucidated functions of this protein family include the mediation of vascular regression, as well as the generation of ECM fragments with antiangiogenic properties.

MMP2 and MMP9 belong to the class of gelatinases with collagen and fibrin as substrates and are expressed as inactive pro-proteins. MMP2 and MMP9 are processed to activate their metalloprotease activity. MT1-MMP is a member of the membrane associated or membrane-type MMPs [7-9]. In the vasculature, MT-MMPs are thought to be involved in the localized pericellular release of bioactive growth factors such as bFGF, VEGF, and TGF-beta, processing of pro-MMP2 into an activated MMP2, and degradation of ECM components including collagens and fibrins [10,11]. MT1-MMP has been shown to be the most potent fibrinolytic MMP and has a critical role in the creation of channels in the ECM, into which endothelial cells migrate during sprouting angiogenesis [12,13]. MT1-MMP function is also required for proper lumenization of the new vessel [13].

Notch signaling is an evolutionarily conserved pathway that regulates cell fate decisions. Notch proteins, Notch 1 through 4, act as receptors and their ligands Jagged (Jagged-1, 2) and Delta-like (Dll-1, 3, 4), are all transmembrane proteins [14]. Upon ligand binding, the cytoplasmic domain of Notch is released by proteolytic cleavage via presenilin/γ-secretase [15], translocates to the nucleus, and interacts with the transcriptional repressor CSL (CBF1/Su(H)/Lag2), converting it to a transcriptional activator [16]. An essential role for Notch signaling in arterial differentiation and vascular remodeling has been demonstrated by genetic studies of mice with targeted mutations in either Notch (*Notch1^-/-^*, and *Notch1^-/-^*;*Notch4^-/-^*), or Notch ligands (*Jagged1^-/- ^*and *Dll4^-/+^*or *Dll4^-/-^*), reviewed in Shawber et al. [14]. In addition, targeted activation of Notch4 specifically in endothelial cells disrupted vascular remodeling, resulting in embryonic lethality [17]. These studies demonstrate that proper levels of Notch signaling are essential for patterning of the vasculature during a period of embryonic development that is known to be critically dependent on VEGF [14,18].

Signaling via both VEGF and Notch is indispensable in vascular development, and it has become evident that these two pathways are interconnected. Heterozygous deficiency of *Dll4 *results in embryonic lethality with profound vascular defects, including defective arterial branching from the aorta and arterial regression, processes that also depend on VEGF signaling [19-21] suggesting Dll4 and VEGF work in concert. In cultured human arterial endothelial cells, VEGF, but not bFGF, induced expression of Notch1 and Dll4 [22]. Furthermore, expression of Dll4 reduced VEGF/VEGFR-2 signaling, likely via downregulation of VEGFR-2 expression in cultured endothelial cells [23]. A role for Notch signaling in tumor angiogenesis was originally hypothesized from the observation that VEGF induced Dll4 in the angiogenic endothelium of tumor xenografts [24] and blocking Dll4 functions resulted in dysregulated non-productive angiogenesis [24,25].

In this study, we demonstrate that Notch mediates VEGF-induced MMP activity in endothelial cells. A Notch antagonist, called Notch1 decoy, blocked VEGF activation of Notch/CSL signaling, VEGF-induced HUVEC morphogenesis on both collagen and fibrin gels, and VEGF-induced fibrinolysis. Notch signaling upregulated the expression of MMP9 and MT1-MMP, and activated MMP2 and MMP9 in endothelial cells. Accordingly, we found that the Notch1 decoy-mediated suppression of HUVEC morphogenesis occurred via inhibition of MMP activity. Finally, Notch1 decoy suppressed endothelial MMP9 expression in an *in vivo *neovascularization model in mice. These data demonstrate that Notch directly regulates the endothelial cell response to VEGF via induction of MMPs.

## Methods

### Reagents and Expression Vectors (adenovirus/retrovirus)

GM6001 was used at 50 μM (Elastin Products Company). eACA was used at 10 mM (Sigma). SU5416 is from Eisai Co. LTD. Notch1 decoy (N1decoy) encodes the extracellular domain of rat Notch1 (bp 241-4229, accession# X57405) fused in frame to human IgG Fc, as described [26]. The constitutively active Notch1 adenovirus (N1IC) encodes the cytoplasmic domain of human Notch1 as described [27]. LacZ, human VEGF^165^, and Notch1 decoy cDNAs were engineered into pAdlox, recombinant adenoviruses generated and stocks produced as described [28].

### Cells and Adenoviral Infections

HUVEC were isolated from human umbilical vein as described [29] and grown using EGM-2 Bullet kit (complete medium, LONZA). Porcine type I collagen is from Nitta Gelatin (Osaka, Japan). Fibrin gels were made by combining 2 mg/ml fibrinogen (Sigma) and 0.0625 U/ml thrombin (Sigma) at 4°C, followed by a one-hour incubation at 37°C. Adenovirus infections were as described [30] at indicated dosage (expressed as MOI - multiplicity of infection).

### Western Blotting

HUVEC were cultured on type I collagen-coated plates for 5 days in complete media, then starved in serum free media (SFM, Invitrogen) for 48 hours and cell lysates collected in TENT buffer (50 mM Tris pH8.0, 2 mM EDTA, 150 mM NaCl, 1% Triton X-100). Westerns were conducted using an anti-MT1-MMP antibody (Ab-1, EMD Biosciences).

### RT-PCR

HUVEC were seeded on type I collagen gels 2 days after infection and cultured post-confluence for 5 days before isolation of total RNA. Total RNA was isolated with RNeasy mini kit (Qiagen) and First-strand cDNA was synthesized using SuperScript II™ (Invitrogen). PCR primers were designed to recognize transcripts for GAPDH, Jagged1, Dll4, Notch1, Notch4, MMP2, MMP9, MT1-MMP and MT2-MMP. PCR used Platinum Taq DNA polymerase (Invitrogen) and reactions removed at noted cycle number and product analyzed as described [30]. The following oligos were used for RT-PCR:

MT1-MMP

(5'-CGCTACGCCATCCAGGGTCTCAAA-3', 5'-CGGTCATCATCGGGCAGCACAAAA-3'),

MT2-MMP

(5'-TCGACGAAGAGACCAAGGAG-3',

5'-ACTGCCACCAGGAAGAGGTT-3'),

MMP2

(5'-GGGACAAGAACCAGATCACATAC-3',

5'-CTTCTCAAAGTTGTAGGTGGTGG-3'),

MMP9

(5'-GTATTTGTTCAAGGATGGGAAGTAC-3', 5'-GCAGGATGTCATAGGTCACGTAG-3'),

GAPDH

(5'-CGGAGTCAACGGATTTGGTCGTAT-3', 5'-AGCCTTCTCCATGGTGGTGAAGAC-3').

### Luciferase Reporter Assay

HUVEC (3.0×10^5^) were infected with adenovirus encoding human VEGF^165^(Ad-VEGF) and two days later transfected with Effectene (Qiagen) using 475 ng CSL luciferase reporter (pGA981-6) [31] 25 ng Renilla luciferase pRL-SV40 (Promega, Madison, WI) for normalizing transfection efficiency. The following day Luciferase and Renilla activity was determined with Dual-Luciferase^® ^Reporter Assay System (Promega) and Berthold dual-injection luminometer. Notch1 decoy effects on VEGF-activity were evaluated with reporter assays, as above, after co-transduction of HUVEC with adenovirus encoding Notch1 decoy (Ad-N1ECDFc) and Ad-VEGF. All assays were performed in triplicate.

### HUVEC Morphogenesis Assay

HUVEC morphogenesis was assessed via microscopy and scored as cells with single or multiple processes after 5 days of culturing on porcine type I collagen gels, as described [32]. Adenovirus infections were performed 2 days prior to seeding on collagen gels. GM6001 was added to medium 1 hr after HUVEC seeding. Cell number was measured using the Cell Counting Kit-8 (Dojindo Molecular Technologies).

### Fibrinolytic Assay

HUVEC were seeded as a monolayer on bovine fibrin gels in complete medium in 24 well plate. After 5 days, wells were incubated with 330 ng/ml Thiazolyl Blue Tetrazolium Bromide (4) (Sigma) for 2 hours. Reagent was removed and wells documented by digital photography. Experiments were performed in triplicate.

### Gelatin Zymography

Gelatin Zymography was performed on HUVEC conditioned media as described[33]. HUVEC were infected with Ad-VEGF at a MOI of 40 or co-infected with Ad-VEGF and Ad-N1ECDFc each at a MOI of 40. Adenovirus encoding LacZ (Ad-LacZ) was used to normalize infections to 100 MOI. Adenovirus-transduced-HUVEC were cultured on collagen gels, conditioned medium collected at indicated days for MMP9 and day 4 for MMP2.

### Dorsal Air Sac (DAS) Assay

The DAS assay was performed as described [34]. KP1/VEGF121 cells were transduced with Ad-GFP or Ad-Notch1 decoy at 60 MOI and packed into Millipore chambers that were transplanted into a DAS of C57BL/6 mice. Mice were sacrificed four days after implantation, and dorsal skin removed and embedded in OCT. Each group consisted of 3-5 mice, and experiments were done in duplicate. Dermal cross-sections were immunostained as described[26] with antibodies against PECAM (Pharminigen) and MMP9 (ABcam) and visualized and imaged by fluorescent microscopy at 20× magnification.

## Results

### VEGF-induced HUVEC morphogenesis is mediated by Notch

**Figure 1:**
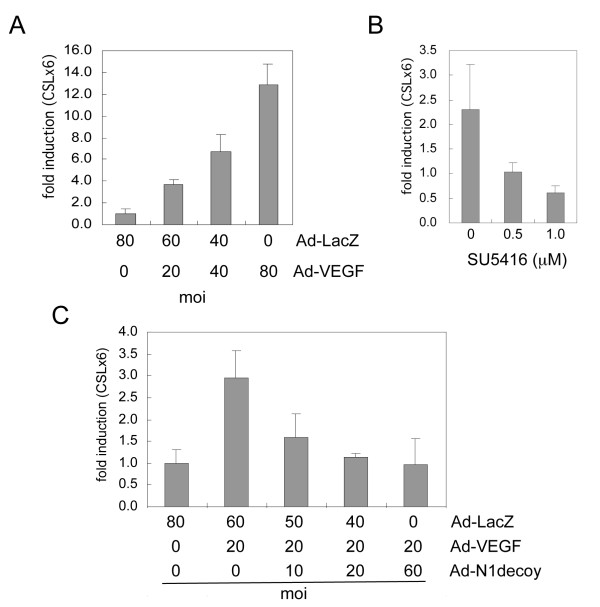
**VEGF activated Notch signaling in HUVEC**. A) HUVEC were transduced with increasing amounts of Ad-VEGF as indicated, and Notch/CSL reporter activity determined. B) The VEGFR inhibitor (SU5416) perturbed Notch signal activation downstream of VEGF. HUVEC were transduced with Ad-VEGF at 40 MOI and increasing amounts of SU5416 added to HUVEC prior to determining CSL reporter activity. C) Notch1 decoy blocked VEGF-induced Notch signal activation. HUVEC were transduced with Ad-VEGF at 20 MOI and increasing amounts of Ad-Notch1 (N1) decoy as indicated. Ad-LacZ was added to transductions to normalize the MOI between conditions. Notch/CSL reporter luciferase data are represented as fold induction relative to Ad-LacZ controls. Experiments were performed in triplicate, and data of a representative experiment presented.

Notch functions downstream of VEGF in arterial/venous specification [35]. We first determined if VEGF induced Notch signaling *in vitro *in human umbilical vein endothelial cells (HUVEC). HUVEC were infected with increasing MOIs of an adenovirus encoding VEGF-A (Ad-VEGF). Two days later, a Notch-responsive CSL-luciferase reporter was introduced by lipofection, and luciferase activity determined the following day [36]. VEGF-transduced HUVEC exhibited Notch signal activation in a dose dependent manner, as measured by the transactivation of the CSL-reporter (Figure 1A). Addition of SU5416, an inhibitor of VEGF receptor kinases (VEGFR-1 and VEGFR-2) [37], decreased VEGF-induced CSL reporter activity (Figure 1B), indicating that the induction of Notch signaling by VEGF depends on VEGF receptor activity. To inhibit Notch signaling in this assay, we infected HUVEC with adenovirus (Ad)-VEGF and increasing amounts of the Notch antagonist, Notch1 decoy (Ad-N1decoy) [26,38], which encodes the extracellular EGF-repeat domain of Notch1 fused to human Fc. Notch1 decoy inhibited VEGF-induced activation of Notch signaling in a dose dependent manner (Figure 1C), demonstrating that VEGF induced ligand-dependent Notch activation. Consistent with this hypothesis, VEGF upregulated expression of Notch4 and the Notch ligand, Dll4 in HUVEC (Additional File 1), similar to a previous report [22]. Jagged1 and Notch1 expression was not significantly altered (Additional File 1). Our data suggests that VEGF via VEGFR signal activation induced Notch signaling in HUVEC by upregulating Notch4 and its ligand, Dll4.

**Figure 2:**
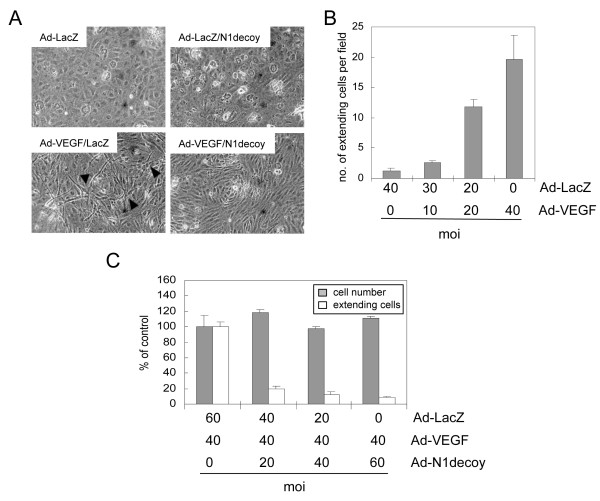
**VEGF-dependent HUVEC morphogenic changes are mediated by Notch**. A) HUVEC were transduced with Ad-LacZ or Ad-VEGF, with or without Ad-Notch1 (N1) decoy at 40 MOI. Ad-LacZ was used to normalize MOI to 80. VEGF induced HUVEC morphogenesis (lower left panel) that was blocked by N1 decoy (lower right panel). B) Quantification of Ad-VEGF-induced morphogenesis, measured as numbers of HUVEC with cellular extensions. HUVEC were transduced with increasing amounts of Ad-VEGF as indicated and Ad-LacZ was used to normalize MOI to 40. C) N1 decoy blocked VEGF-induced HUVEC morphological changes without affecting total cell number. HUVEC were transduced with Ad-VEGF at 40 MOI and increasing amounts of Ad-N1 decoy as indicated. Ad-LacZ was used to normalize the MOI to 100. Experiments were performed in triplicate, and data of a representative experiment presented.

When grown on three-dimensional fibrin or collagen gels, VEGF promotes the formation of cellular extensions that invade the underlying matrix, a phenomenon termed "HUVEC morphogenesis" [39,40]. To determine if Notch acts downstream of VEGF-induced morphogenesis, HUVEC were transduced with Ad-VEGF or Ad-LacZ with or without Ad-Notch1 decoy. Three days after seeding on type I collagen gels, HUVEC were scored for the presence of cellular extensions and cell number determined. VEGF promoted HUVEC morphogenesis on collagen gels (Figure 2A), consistent with previous reports [26,32,39,40]. The number of HUVEC with cellular extensions was dependent on the dose of VEGF provided by adenoviral transduction (Figure 2B). In this assay, adenoviral co-transduction of Notch1 decoy with VEGF inhibited VEGF-induced HUVEC morphogenesis, with a nominal effect on proliferation (Figure 2A, C). Thus, Notch mediates VEGF-induced HUVEC morphogenesis on type I collagen and fibrin gels (Figure 2 and data not shown).

### Notch mediates VEGF-induced fibrinolysis

**Figure 3:**
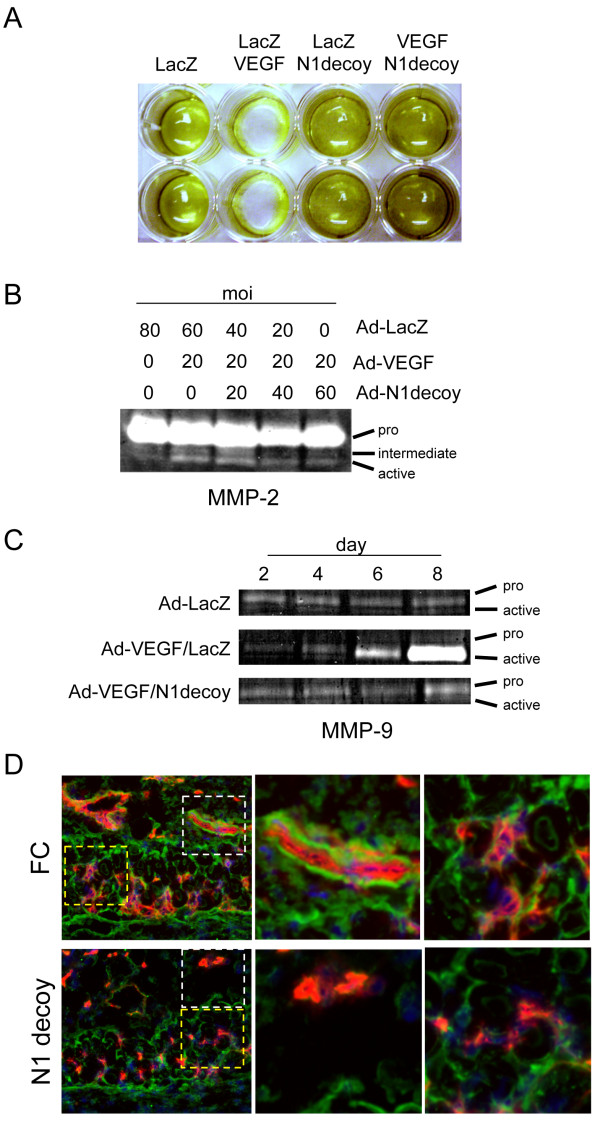
**Notch mediates VEGF-induced fibrinolysis and gelatinase activity**. A) N1 decoy blocked VEGF-induced fibrinolytic activity. HUVEC were transduced with Ad-VEGF, Ad-N1decoy or both at 40 MOI and Ad-LacZ was used as a control and to normalize the MOI to 80. Transductants were seeded on fibrin gels and fibrin degradation determined after 5 days. B) N1decoy blocked VEGF-induced MMP2 activation. HUVEC were transduced with Ad-VEGF at 20 MOI and increasing amounts of Ad-N1decoy as indicated. Ad-LacZ was used as a control and to normalize the MOI to 80. Four days post-infection, supernatants were collected and subjected to gelatinase zymography. C) N1 decoy blocked VEGF-induced expression and activation of MMP9. HUVEC were transduced with Ad-VEGF at 40 MOI, with or without Ad-N1decoy at 40 MOI and Ad-LacZ was used as a control and to normalize the MOI to 80. Supernatants from transduced HUVEC were collected at 2, 4, 6, and 8 days post-infection and subjected to gelatinase zymography. D) Cross-section of skin from DAS assay stained for PECAM (red) and MMP9 (green). White boxes highlight the large vessels within the subcutaneous adipose depot. Yellow boxes highlight the capillaries within the dermal smooth muscle cell layer. Representative photographs are shown.

We next determined if VEGF-mediated ECM degradation occurred via a Notch dependent mechanism. HUVEC were infected with Ad-LacZ or Ad-VEGF with or without Ad-Notch1 decoy and seeded on top of fibrin gels. Six days later, the fibrin gels were incubated with Thiazolyl Blue Tetrazolium Bromide and visualized. Expression of VEGF resulted in the complete degradation of the fibrin gel (Figure 3A). Co-expression of Notch1 decoy with VEGF blocked the fibrinolysis, suggesting that Notch mediates VEGF-induced fibrinolysis in HUVEC.

### Notch regulates VEGF-induced matrix metalloprotease activity

The gelatinases MMP2 and MMP9 function to degrade both fibrin and collagen matrices produced by endothelial cells [41]. VEGF signaling promotes the secretion and activation of MMPs, such as MMP2 and MMP9 [42]. As Notch signaling mediated VEGF-induced HUVEC morphogenesis, we determined if the induction of MMP2 and MMP9 activity by VEGF was mediated by Notch. HUVEC were infected with Ad-VEGF and increasing amounts of Ad-Notch1 decoy, and conditioned media was used in zymographic analysis. After four days, the level of MMP2 pro-form was unchanged, while VEGF converted MMP2 to an active form (Figure 3B). VEGF-induced MMP9 activity 4 days post-transduction, and this activity increased over the following four days (Figure 3C). The VEGF-mediated MMP activation we observed was consistent with previous reports [42]. To examine the role of Notch in the VEGF induced MMP activities, we assessed MMPs after expression of the Notch1 decoy. Co-expression of VEGF with Notch1 decoy inhibited the activation of MMP2 and blocked the transcriptional induction and biochemical activation of MMP9 by VEGF (Figure 3B, C). Thus, VEGF activated MMP2 and MMP9 gelatinase activity by a Notch-dependent mechanism.

To determine if Notch influenced MMP9 expression during VEGF-induced angiogenesis, we evaluated the expression of MMP9 *in vivo *using a dorsal air sac angiogenesis (DAS) model [34]. In the DAS model, a chamber containing VEGF-expressing KP1 cells is implanted in the air sac under the dorsal skin. VEGF is released from the chamber and induces neo-angiogenesis in the overlying skin. Using this model, we have previously demonstrated that Notch1 decoy perturbed VEGF-driven angiogenesis and suppressed endothelial VEGFR-1 expression [43]. To evaluate MMP9 expression in this model, cross-sections of skin from DAS assays using either control VEGF/Fc- or VEGF/Notch1 decoy-expressing KP1 cells were co-immunostained with antibodies against MMP9 and the endothelial cell marker PECAM. In the control Fc-expressing tissues, MMP9 expression was observed around the vessels within both the smooth muscle cell layer and subcutaneous fat depot (Figure 3D). In the Notch1-decoy DAS tissues, there was a reduction in PECAM staining indicating reduced vasculature, as we previously reported [26]. We also observed a loss of endothelial-associated MMP9 expression in the Notch1 decoy-expressing DAS tissues relative to Fc control tissue. Thus, we demonstrate that blocking Notch signaling downstream of VEGF suppressed endothelial MMP9 expression *in vivo*.

### Notch signaling induces MMP9 and MT1-MMP expression in HUVEC

**Figure 4:**
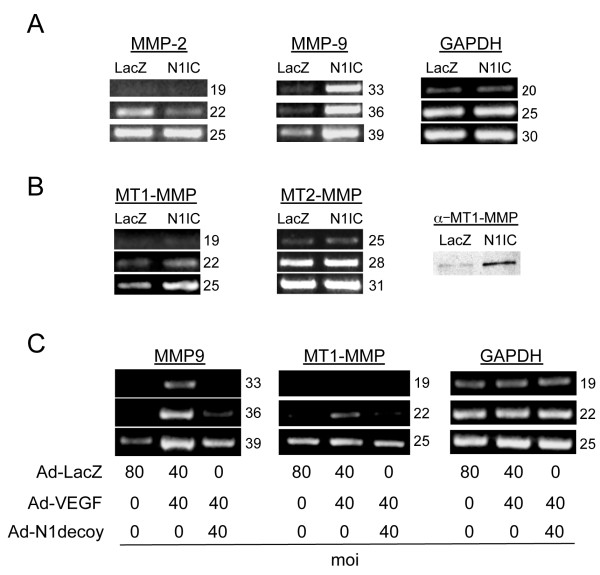
**Notch induced MMP9 and MT1-MMP expression downstream of VEGF**. A) Transduction of an activated form of Notch1 (N1IC) induced MMP9 transcripts in HUVEC. RT-PCR analysis of MMP2 and MMP9 compared to control GAPDH. B) Transduction of N1IC induced MT1-MMP. RT-PCR analysis of MT1-MMP and MT2-MMP (left panels) compared to control GAPDH (see A). Western analysis of MT1-MMP (right panel). C) N1decoy blocked VEGF-mediated induction of MMP9 and MT1-MMP determined by RT-PCR; GAPDH serves as a control. HUVEC were transduced with Ad-VEGF at 40 MOI, with or without Ad-N1decoy at 40 MOI and Ad-LacZ was used as a control and to normalize the MOI to 80. For RT-PCR experiments, the number of PCR cycles is indicated to the right of the figures. Experiments were performed in triplicate, and data of a representative experiment presented.

Since inhibition of Notch signaling abrogated VEGF induction of MMP2 and MMP9 activity, we next examined whether Notch activation upregulated the expression of MMP2 and MMP9 in HUVEC. HUVEC were infected with an adenovirus encoding a constitutively active cytoplasmic form of Notch1 (N1IC), RNA isolated and RT-PCR performed for MMP2, MMP9, and GAPDH. Notch1 activation upregulated MMP9 transcripts (Figure 4A). Though we found that Notch1 decoy blocked VEGF from activating MMP2 (Figure 3B), increased Notch1 signaling paradoxically suppressed MMP2 transcript levels (Figure 4A). On the cell surface, MMP2 is activated by membrane-type MMPs (MT-MMPs) [7]; therefore, we determined if Notch signaling altered the expression of MT-MMPs, MT1-MMP and MT2-MMP. Expression of the activated form of Notch1 upregulated both MT1-MMP transcripts and protein, but had no affect on MT2-MMP (Figure 4B).

Next, we asked whether Notch activity is required for the VEGF-induction of MMP9 and MT1-MMP. Ectopic expression of VEGF increased both MMP9 and MT1-MMP transcripts (Figure 4C). Co-infections of Ad-Notch1 decoy with Ad-VEGF suppressed the expression of both MMP9 and MT1-MMP transcripts. Taken together, these data demonstrate that Notch1 activation increased MMP2 and MMP9 activity. Thus, Notch functions downstream of VEGF to regulate MMPs via distinct mechanisms; Notch upregulated MMP9 activity at the transcriptional level, while it regulated MMP2 activity at the cell surface via the induction of MT1-MMP.

### Notch-induced HUVEC morphogenesis is mediated by MMPs

**Figure 5:**
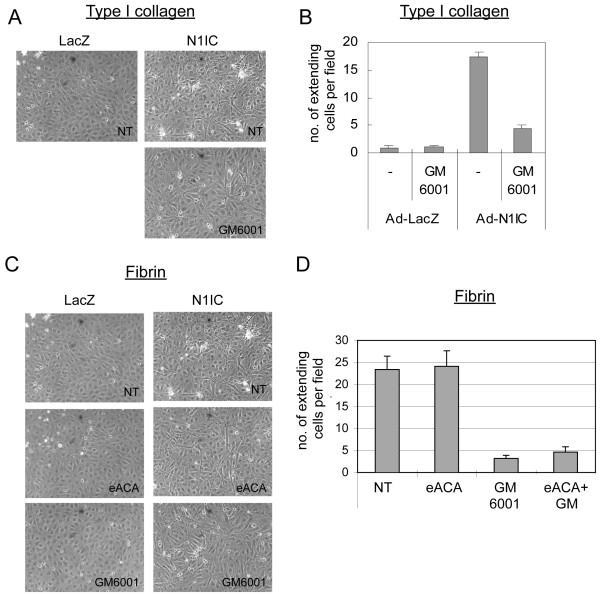
**Notch-induced HUVEC morphogenesis was mediated via its induction of MMPs**. A) Notch-induced HUVEC morphogenesis on collagen was blocked by addition of an MMP inhibitor. HUVEC were transduced with either Ad-LacZ or Ad-N1IC at 40 MOI and seeded on type 1 collagen gels in the absence or presence of 50 μM GM6001. Images are representative of 5 days post-infection. B) Quantification of Ad-N1IC-induced morphogenesis on collagen, measured as numbers of HUVEC with cellular extensions. C) Notch-induced HUVEC morphogenesis on fibrin was blocked by addition of an MMP inhibitor, but not a serine-protease inhibitor. HUVEC were transduced with either Ad-LacZ or Ad-N1IC at 40 MOI and seeded on fibrin gels in the absence or presence of the matrix metalloprotease inhibitor (50 μM GM6001), or the serine-protease inhibitor, (10 mM eACE). Images are representative of 5 days post-infection. D) Quantification of Ad-N1IC-induced morphogenesis on fibrin. Experiments were performed in triplicate, and data of a representative experiment presented. NT (No Treatment).

Since VEGF-induced HUVEC morphogenesis requires Notch signal activation, we determined whether Notch mediates this process via MMP activation. HUVEC were transduced with Ad-LacZ or Ad-N1IC and seeded on type I collagen or fibrin gels. After 7 days, the presence of HUVEC with cell extensions was evaluated and quantified. We used an adenovirus that co-expressed N1IC and GFP to visualize the cells expressing the activated form of Notch1. Notch1 activation induced HUVEC morphological changes (Additional File 2), similar to that observed for ectopic VEGF (Figure 2A). In this assay, Notch1 functioned in a cell autonomous fashion, as Notch-activated cells (identified as GFP positive cells) displayed cellular extensions when compared to GFP negative cells. Moreover, the number of HUVEC undergoing morphological changes increased with higher doses of Ad-N1IC adenovirus (data not shown). Using this assay, we asked if Notch promoted HUVEC morphogenesis via the induction and activation of MMPs. Ad-LacZ and Ad-N1IC-transduced HUVEC were seeded on either type I collagen or fibrin gels and the MMP inhibitor GM6001 introduced to the media. GM6001 is an inhibitor that targets multiple MMPs, including MMP2, MMP9, and MT1-MMP [44]. Notch-mediated HUVEC morphogenesis was inhibited by GM6001 when assayed on either collagen (Figure 5A, B) or fibrin gels (Figure 5C,D). As fibrinolysis also involves the uPA/PAR pathway, we introduced the serine-protease inhibitor, eACE, to block this pathway in Ad-infected HUVEC grown on fibrin gels (Figure 5C, D). The serine-protease inhibitor did not block Notch induced HUVEC morphogenesis, and did not have an additive affect when used in combination with the MMP inhibitor GM6001 (Figure 5D). Thus, VEGF requires Notch signaling to regulate both expression and activation of specific MMPs, and Notch, in turn, utilizes MMP activation to promote HUVEC morphogenesis.

## Discussion

In this study, we describe a new means by which Notch functions downstream of VEGF to regulate the angiogenic process. We identified MMPs as novel targets of Notch signaling in endothelial cells. Our data suggest that VEGF activates Notch signaling by upregulating the expression of Dll4 and Notch4, consistent with other reports [22]. Notch1 decoy blocked VEGF-activation of Notch/CSL signaling, VEGF-induced HUVEC morphogenesis on collagen and fibrin gels, VEGF-dependent fibrinolysis and VEGF-induced dermal angiogenesis *in vivo*. Notch signaling, in turn, mediated VEGF-induced matrix metalloprotease activity by upregulating the expression of MMP9 and MT1-MMP, likely leading to activation of MMP9 and MMP2. These MMPs function as key regulators of angiogenesis in both physiological and pathological settings [45]. During angiogenesis, MMP activity functions to promote ECM degradation, lumen formation, the activation of membrane receptors, and release of ECM/membrane-associated pro-angiogenic growth factors, such as VEGF, bFGF and TGF-β [4]. MMPs also have anti-angiogenic functions, as they are capable of generating matrix protein fragments that suppress angiogenesis [46]. Taken altogether, we propose that Notch signaling functions downstream of VEGF to affect angiogenesis in part by inducing endothelial cell localized matrix metalloprotease activity.

Pericellular matrix metalloproteases regulate the local availability of bioactive angiogenic factors, such as VEGF and bFGF. It has been proposed that matrix metalloproteases function to increase VEGF activity to promote pathological angiogenesis during tumorigenesis [42]. Consistent with this hypothesis, deletion of MMP9 in a mouse model of pancreatic β-cell carcinoma, Rip1Tag2, suppressed tumor progression [47]. In these mice, the level of VEGF was unaltered, but mobilization of VEGF from the extracellular matrix was inhibited. Thus, endothelial upregulation of MMP9 by Notch may lead to the localized release and activation of matrix-bound growth factors.

Besides its role in processing and activating MMP2, MT1-MMP also functions to degrade the surrounding fibrin matrix during physiological and pathological angiogenesis. The breakdown of the local fibrin matrix is essential in the initiation and propagation of angiogenic responses, and generates a provisional matrix that can sustain the formation of nascent vessels during wound healing, inflammation and tumor growth. MT1-MMP localizes at the leading edge of migrating endothelial cells and is thought to aid in the degradation of the extracellular matrix to facilitate endothelial cell invasion [48]. Although MT1-MMP may be the most potent fibrinolytic MMP, the gelatinases MMP2 and MMP9 also recognize fibrin as a substrate. More recently, Stratman et al demonstrated that the formation of vascular guidance tunnels within collagen gels occurred via a MT1-MMP-dependent proteolytic process and that these tunnels become conduits for EC motility in sprouting angiogenesis [13] Thus, the induction of MT1-MMP by Notch may lead to the activation of MMP2 that together with MT1-MMP and MMP9 promote extracellular matrix degradation and endothelial cell morphogenesis.

MT1-MMP activity has also been shown to be critical for endothelial migration during sprouting angiogenesis [42,49]; however Notch signal activation is a potent inhibitor of endothelial cell migration [50,51], which suggests that Notch signaling has both anti- and pro-angiogenic functions. Thus, Notch may have an early pro-angiogenic function in the induction and activation MMPs to promote the degradation of the pericellular matrix, but it subsequently needs to be turned off to allow for endothelial cell migration and tube formation to progress. MT1-MMP activity has also been shown to be critical for endothelial lumen formation [13]. Taking this finding into consideration, Notch may be turned off in tip cells and induces MMPs in stalk cells, which may be important for remodeling the matrix during the process of lumen formation. The latter model fits well with our current understanding of Notch function in the restriction of endothelial sprouting by blocking tip cell differentiation [52].

The molecular mechanism by which Notch regulates MMP9 and MT1-MMP expression in HUVEC is unclear. In lung metastases of Rip1Tag2 mice, MMP9 expression, within the endothelium, was regulated by VEGFR-1 [53]. Since Notch induces VEGFR-1 expression in endothelial cells [43,54], VEGFR-1 signaling may mediate the induction of MMP9 by Notch. However, Notch1 has also been shown to regulate the expression and activation of MMP9 through NF-κB in pancreatic cancer cells [55], suggesting that the regulation of MMP9 expression and activity in endothelial cells by Notch may be mediated by NF-κB signaling. Finally, the activation of MMP9 at the cell surface is dependent on the uPA/plasmin pathway. In human prostatic cancer cells, knock-down of Notch1 resulted in a downregulation of MMP9 and uPA and its receptor, uPAR [56]. From our data it is unclear whether Notch regulates MMP9 solely at the level of transcription, and the possibility remains that it may regulate the uPA/plasmin pathway to activate MMP9 at the cell surface. This is a subject of future investigation.

We propose that Notch activates MMP2 via induction of MT1-MMP, which is present in its active form at the cell surface. In the tumor microenvironment, an imbalance may arise from an increase in MMP expression/activity and a decrease in the expression of the MT-MMP inhibitors, TIMP-2 or TIMP-3 [4,5]. This imbalance would result in a pro-angiogenic state. A similar imbalance in MMP/TIMP activity ratio has also been implicated in aortic aneurysm, varicose veins, hypertension and preeclampsia [6]. As inhibition of Notch signaling with Notch1 decoy blocked the induction of MMP9 and MT1-MMP transcripts in HUVEC, blocking Notch signaling in pathological settings may perturb angiogenesis and may prove therapeutically useful in the treatment of vascular disorders.

## List of Abbreviations

VEGF: vascular endothelial growth factor; VEGFR: vascular endothelial cell growth factor receptor; Dll: Delta-like; HUVEC: human umbilical vein endothelial cell; MMP: matrix metalloprotease; MT-MMP: membrane type matrix metalloprotease; ECM: extracellular matrix; MOI: multiplicity of infection; TIMP: tissue inhibitor of metalloproteases

## Competing interests

The authors declare that they have no competing interests.

## Authors' contributions

YF participated in the design of the studies, carried out the HUVEC studies, conducted the dorsal air sac assay, and drafted the manuscript. CJS participated in the design of the studies, conducted immunohistochemical analyses and co-wrote the manuscript. AS assisted in experiments using adenovirus, HUVEC cultures, and zymography. EK assisted in *in vitro *expression analysis experiments. YKC carried out immunohistochemistry studies. JK conceived of the study and participated in its design and coordination and edited the final manuscript. All authors read and approved the final manuscript.

## Supplementary Material

Additional file 1 **VEGF induced Notch and Notch ligand expression in HUVEC**. HUVEC were transduced with either Ad-LacZ or Ad-VEGF at 40 MOI. Two days later, total RNA was isolated and RT-PCR performed with PCR primers designed to amplify GAPDH, Notch1, Notch4, Jagged1, Dll4. Reactions were removed at noted cycle number and product analyzed as described [30]. Number of PCR cycles is indicated. Click here for file

Additional file 2 **Notch signal activation cell autonomously altered HUVEC morphology**. HUVEC were transduced with an adenovirus which co-expresses N1IC and GFP at 40 MOI. N1IC/GFP expressing tranductants were mixed with control HUVEC and cultured on type 1 collagen gels for 7 days. Notch activated cells, identified as GFP positive cells, display cellular extension, as compared to GFP negative cells. Click here for file
