# Supplementary figures and images for: Notch modulates VEGF action in endothelial cells by inducing Matrix Metalloprotease activity
Source: Vasc Cell. 2011 Jan 18;3:2. doi: 10.1186/2045-824X-3-2 (PMC3039832; doi:10.1186/2045-824X-3-2)

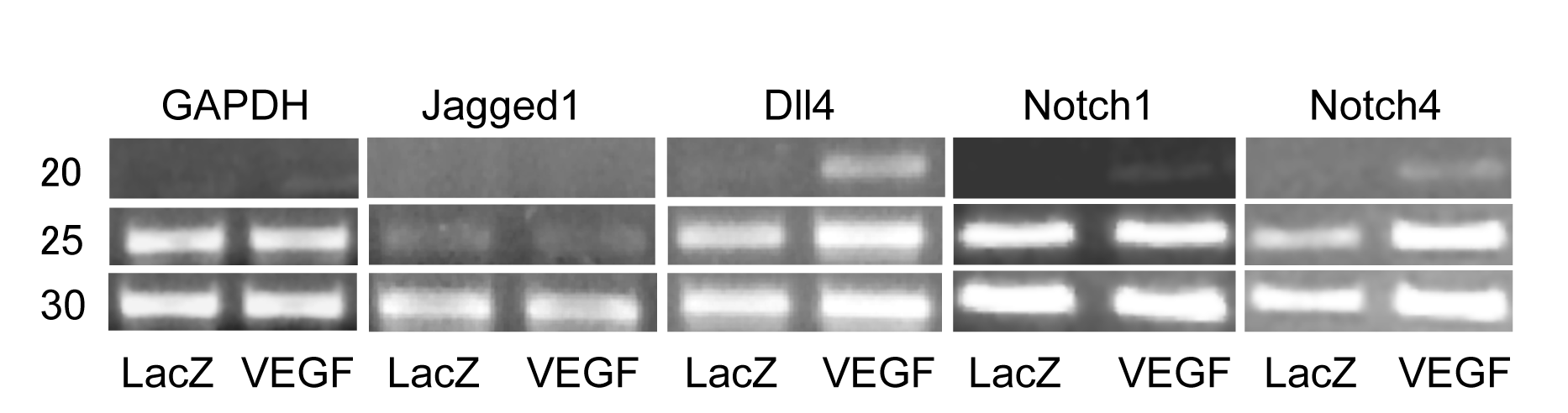

Supplement: Additional file 1 — VEGF induced Notch and Notch ligand expression in HUVEC. HUVEC were transduced with either Ad-LacZ or Ad-VEGF at 40 MOI. Two days later, total RNA was isolated and RT-PCR performed with PCR primers designed to amplify GAPDH, Notch1, Notch4, Jagged1, Dll4. Reactions were removed at noted cycle number and product analyzed as described [30]. Number of PCR cycles is indicated. [file 2045-824X-3-2-S1.TIFF]

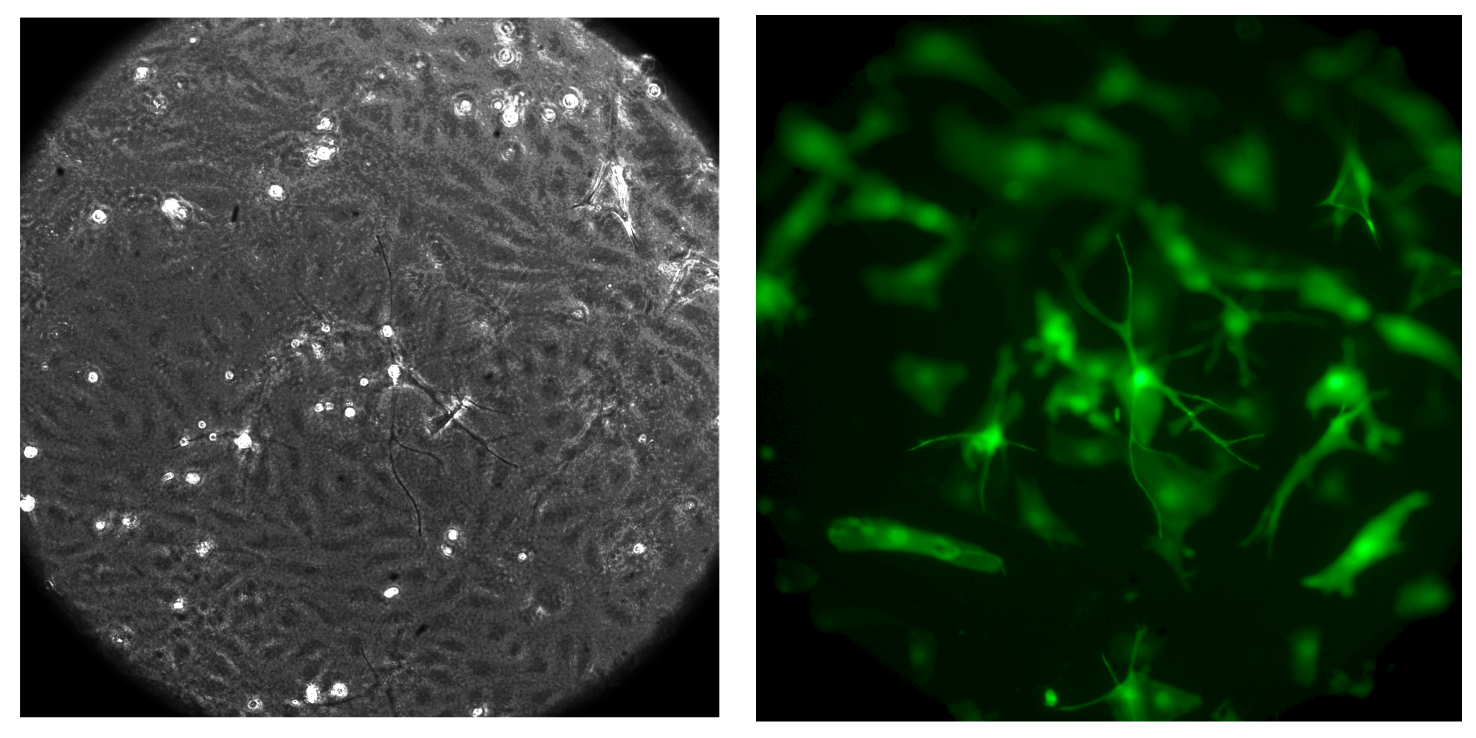

Supplement: Additional file 2 — Notch signal activation cell autonomously altered HUVEC morphology. HUVEC were transduced with an adenovirus which co-expresses N1IC and GFP at 40 MOI. N1IC/GFP expressing tranductants were mixed with control HUVEC and cultured on type 1 collagen gels for 7 days. Notch activated cells, identified as GFP positive cells, display cellular extension, as compared to GFP negative cells. [file 2045-824X-3-2-S2.TIFF]
